# Triple Pancreatic Walled-off Fluid Collections Treated Simultaneously with Endoscopic Transmural Drainage

**DOI:** 10.7759/cureus.2047

**Published:** 2018-01-09

**Authors:** Sameen Khalid, Aamer Abbass, Eric Nellis, Shashin Shah, Hiral Shah

**Affiliations:** 1 Internal Medicine Residency, Florida Hospital-Orlando; 2 Gastroenterology, Lehigh valley health network

**Keywords:** pancreatitis, endoscopy, pancreatic fluid collections, cyst gastrostomy, cyst duodenostomy

## Abstract

Pancreatic pseudocysts and walled-off pancreatic necrosis arise as a complication of pancreatitis. Multiple fluid collections are seen in 5-20% of the patients who have walled-off peripancreatic fluid collections. There is a paucity of data regarding the role of endoscopic transmural drainage in the management of multiple pancreatic fluid collections. In this case report, we present the case of a 72-year-old male with three walled-off pancreatic fluid collections in the setting of acute necrotizing pancreatitis. The patient underwent simultaneous endoscopic ultrasound-assisted cyst gastrostomy and cyst duodenostomy and aggressive irrigation without index endoscopic necrosectomy of the three peripancreatic fluid collections. Significant improvement in the size of the fluid collections was seen on the computed tomography scan, as well as a remarkable immediate clinical improvement after 24 hours of the endoscopic intervention.

## Introduction

Walled-off pancreatic fluid collections (PFCs) are fluid collections that arise as a consequence of pancreatic injury and complicate both acute and chronic pancreatitis. Walled-off PFCs include pseudocysts and walled-off pancreatic necrosis (WOPN). The disruption of the pancreatic duct or side branches results in the collection of fluid that may or may not contain debris [1]. Pancreatic pseudocysts are PFCs surrounded by fibrous tissue without a lining of the epithelium. Secondary infection of the pseudocysts can lead to WOPN formation. WOPN is an encapsulated collection of pancreatic necrosis that contains purulent fluid and necrotic debris and is surrounded by fibrous tissue. Conservative management with periodic observation should be considered for asymptomatic and mildly symptomatic patients. Worsening of symptoms and expansion of fluid collections on imaging should prompt intervention [2]. Treatment of symptomatic patients includes percutaneous radiologic, surgical, and endoscopic drainage [1, 3-4]. Endoscopic drainage can involve cyst gastrostomy, cyst duodenostomy, and transpapillary approaches. In patients with walled-off peripancreatic fluid collections, only 5-20% have multiple fluid collections [5-6]. These patients are typically more symptomatic than patients with a solitary fluid collection and less likely to regress spontaneously [4, 6]. Previously, these patients were candidates for surgical drainage. Now, with increasing expertise in endoscopic techniques and advancement in endoscopic equipment, the role of endoscopic drainage in multiple PFCs needs to be explored. Simultaneous endoscopic transmural drainage of three peripancreatic fluid collections has not been previously reported. We present the case of a 72-year-old patient with three walled-off PFCs arising as a complication of acute pancreatitis. The patient underwent simultaneous endoscopic ultrasound (EUS)-assisted cyst gastrostomy and cyst duodenostomy of the three WOPNs. Significant improvement in the size of the fluid collections was seen on the computed tomography (CT) scan, as well as a remarkable immediate clinical improvement after 24 hours of the endoscopic intervention.

## Case presentation

**Figure 1:**
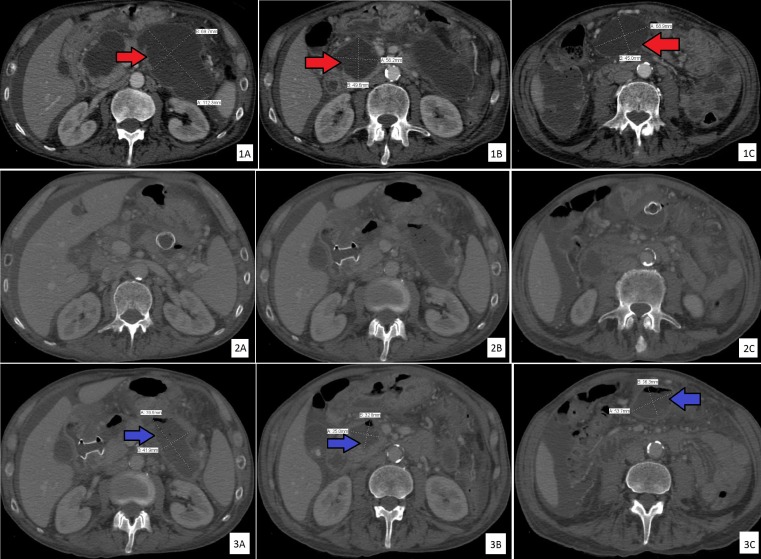
Computed tomography scans of the abdomen Computed tomography scans of the abdomen showing: (1A-C) three peripancreatic fluid collections with enhancing walls before the drainage (red arrows); (2A-C) stents placed during endoscopic transmural drainage; and (3A-C) decreased size of peripancreatic fluid collections on computed tomography scans obtained 24-hours post-endoscopic drainage (blue arrows).

A 72-year-old male presented to an outside hospital with epigastric pain, nausea, and vomiting. Physical examination revealed epigastric tenderness. Laboratory evaluation showed leukocytosis and elevated lipase (1,130 U/L) and amylase (1,442 U/L) with normal aspartate transaminase, alanine transaminase, and alkaline phosphatase. Initial imaging evaluation by ultrasound, computed tomography (CT) of the abdomen, and magnetic resonance cholangiopancreatography showed cholelithiasis with evolving necrotizing pancreatitis and normal intrahepatic, common bile, and pancreatic ducts without evidence of choledocholithiasis. Conservative management was initiated for gallstone pancreatitis; however, the patient failed to improve and was subsequently transferred to our tertiary care center. The patient’s abdominal pain worsened, and he was unable to tolerate anything by mouth due to early satiety, nausea, and vomiting. CT scan of the abdomen revealed a 9.9 x 5.7 cm fluid collection involving the pancreatic body and tail and a 5.2 x 3.6 cm fluid collection along the pancreatic head. Total parenteral nutrition was started. On Day 11, the patient’s clinical condition deteriorated with fever, leukocytosis, and increased epigastric pain. Repeat CT abdomen showed an increased size of peripancreatic fluid collections, which now appeared more well-defined with rim enhancing walls. Mild to moderate ascites was also noted. The largest collection along the pancreatic body and tail, previously measured 9.9 x 5.7 cm, was now 11.2 x 7.0 cm (Figure 1A). The smaller fluid collection along the pancreatic head increased to 5.6 x 5.0 cm from 5.2 x 3.6 cm (Figure 1B). A new collection in the root of the mesentery measuring 6.9 x 4.5 cm was also noted (Figure 1C).

**Figure 2:**
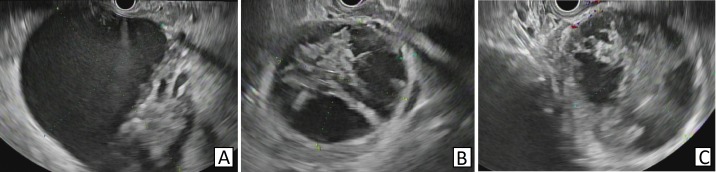
Endoscopic ultrasound A-C: Endoscopic ultrasound showing three hypoechoic peripancreatic fluid collections with debris inside.

Endoscopic ultrasound (EUS) with color Doppler was performed and it showed complex peripancreatic fluid collections with debris inside (Figure 2).

**Figure 3:**
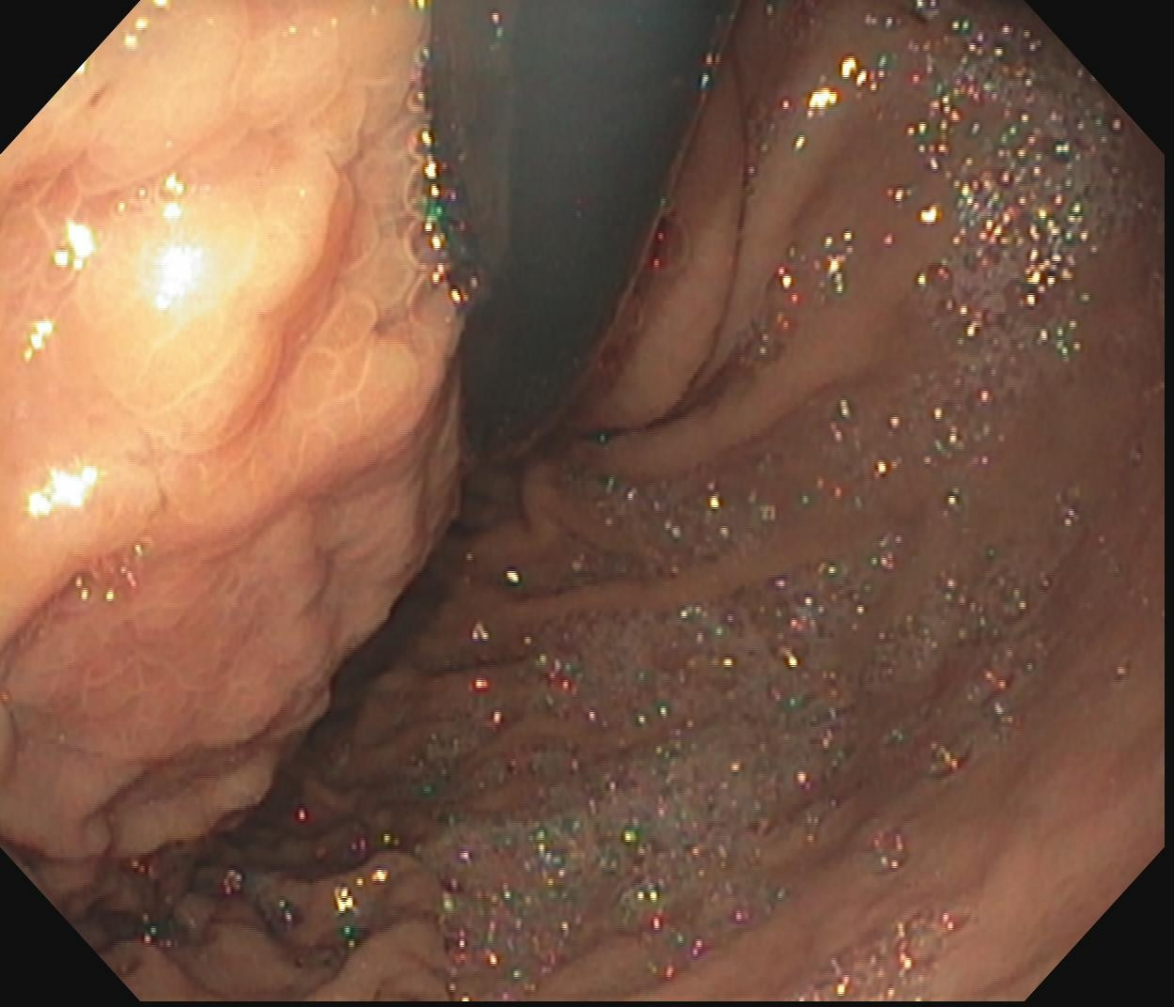
Esophagogastroduodenoscopy showing noticeable bulge from the gastric wall in the proximal stomach that corresponded to one of the pseudocysts on computed tomography scan.

EUS confirmed that the WOPN were adjacent to the stomach and duodenum with the distance measuring less than 1 cm and there were no associated pseudoaneurysms. There was no communication of the PFCs with the pancreatic duct. Esophagogastroduodenoscopy (EGD) showed esophagitis in the mid-esophagus, likely from reflux related to partial gastric outlet obstruction from the cysts compressing the stomach. Noticeable bulges from the gastric wall in the proximal stomach and antrum were seen corresponding to the fluid collections seen on the CT scan. Duodenal bulb compression related to another cyst was also seen (Figure 3).

**Figure 4:**
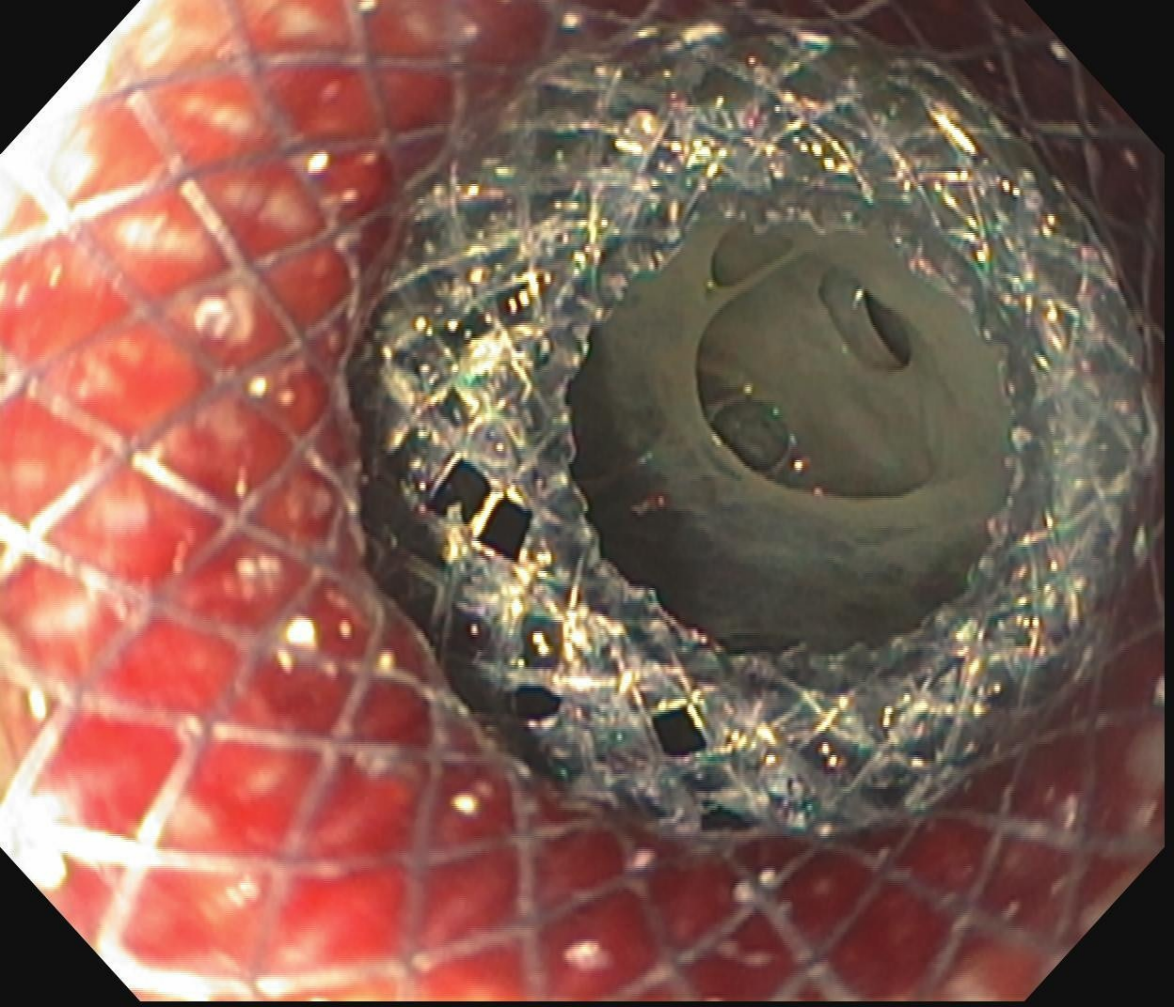
Drainage of walled-off pancreatic necrosis through the stent placed during endoscopic transmural drainage.

EUS demonstrated two cystic collections adjacent to the gastric lumen and one cystic collection adjacent to the duodenal lumen. The patient underwent simultaneous EUS-assisted cyst gastrostomy and cyst duodenostomy using three lumen apposing metal stents (Hot AXIOS™, Boston Scientific, Marlborough, MA) (Figure 1, row 2A-C). Two cystic collections were drained in the gastric lumen and one in the duodenal bulb with drainage of pus and necrotic debris (Figure 4).

Afterwards, all the stents were dilated with through-the-scope (TTS) controlled radial expansion (CRE) dilators. The collections were copiously irrigated with sterile water on index EUS intervention with plans for follow-up repeat necrosectomy. This was followed by rapid improvement in the patient’s symptoms and discontinuation of total parenteral nutrition (TPN) within 72 hours as his diet was eventually progressed from clear liquids to solids. CT of the abdomen 24 hours post-endoscopic management showed the decreased size of peripancreatic fluid collections (Figure 1, row 3A-C). A repeat serum lipase level was normalized (81 U/L). The patient was discharged five days post-procedure in stable condition.

## Discussion

Pancreatic pseudocysts and walled-off pancreatic necrosis are peripancreatic fluid collections that arise as a consequence of pancreatic injury in acute and chronic pancreatitis. In acute pancreatitis, pseudocysts are inflammatory fluid collections surrounded by fibrous or granulation tissue. In chronic pancreatitis, pseudocysts occur in the setting of pancreatic ductal obstruction followed by disruption of the pancreatic duct as a result of increased intraductal pressure [7]. Secondary infection of these pseudocysts leads to walled-off pancreatic necrosis which is more common in acute necrotizing pancreatitis. WOPN is a collection of purulent material with little or no debris. Pseudocysts or WOPN may lead to compression of surrounding structures, such as the stomach, duodenum, and common bile duct, leading to obstruction. Presence of symptoms (pain, early satiety), expansion of fluid collection, and complications (biliary obstruction, infection, rupture, hemorrhage) are indications for drainage [1, 3]. These fluid collections should only be punctured when the wall has matured and after pseudoaneurysm has been ruled out by imaging [2].

Symptomatic PFCs can be drained by percutaneous, surgical, and endoscopic techniques [1, 3-4]. Endoscopic drainage can involve cyst gastrostomy, cyst duodenostomy, and transpapillary approaches. No prospective randomized trials have been performed as of yet to compare the results of the three traditional approaches [3]. The choice of the approach depends on the general condition of the patient, the anatomic relationship of the collection to the stomach and duodenum, the size of the collection, and presence of communication of the pseudocyst with the pancreatic duct [1, 3]. EUS-guided transmural drainage is less invasive than surgery and avoids complications related to percutaneous drainage; hence, it should be given the first preference [5]. In expert hands, endoscopic drainage has shown a success rate of > 90%, a recurrence rate of 10-20%, a mortality rate of 0.2%, and complications in 20% of the cases [4]. Rapid resolution of symptoms, immediate resumption of enteral nutrition, early discharge from the hospital, as well as having one index endoscopic procedure prior to discharge in our patient with complex necrotizing pancreatitis points towards cost-effectiveness and appropriate resource utilization in cases where endoscopic drainage is utilized for multiple pancreatic fluid collections.

Catalano, et al. performed simultaneous endoscopic drainages of multiple pancreatic pseudocysts in their case series of six patients (Catalano M, et al.: Endoscopic drainage of patients presenting with multiple pancreatic pseudocysts (abstract). Pancreat Disord. 1999, 98:A1115). Each of the six patients had two pseudocysts. Half of the pseudocysts were drained via cyst gastrostomy and the other half were drained via cyst duodenostomy. No complications were seen in the cyst gastrostomy group. One patient from the cyst duodenostomy group developed a superinfection and required surgical drainage and debridement. They concluded that treatment of multiple pancreatic pseudocysts by endoscopic drainage is a safe and effective option.

A retrospective study of 165 patients with pancreatic pseudocysts was conducted by Sharma, et al. [8] over a period of 22 years. They concluded that the majority of pancreatic pseudocysts and walled-off pancreatic necrosis can be managed by endoscopic drainage without necrosectomy with a high-resolution rate, lower recurrence rates, and lower morbidity and mortality.

## Conclusions

Our case illustrates that simultaneous endoscopic drainage of up to three pancreatic walled-off fluid collections is a safe, effective, and minimally invasive alternative to surgical drainage in fluid collections with a mature wall within 1 cm of the gastrointestinal lumen. This approach may result in appropriate resource utilization including a decreased length of hospital stay and duration of TPN use. Further large-scale prospective studies are needed to establish the use of endoscopic drainage for the simultaneous management of multiple walled-off pancreatic fluid collections.

## References

[1] Babich JP, Friedel DM (2010). Endoscopic approach to pancreatic pseudocysts: an American perspective. World J Gastrointest Endosc.

[2] Samuelson AL, Shah RJ (2012). Endoscopic management of pancreatic pseudocysts. Gastroenterol Clin North Am.

[3] Pitchumoni CS, Agarwal N (1999). Pancreatic pseudocysts. When and how should drainage be performed?. Gastroenterol Clin North Am.

[4] Săftoiu A, Dumitrescu D, Stoica M (2007). EUS-assisted rendezvous stenting of the pancreatic duct for chronic calcifying pancreatitis with multiple pseudocysts. Pancreatology.

[5] Bikhchandani J, Suraweera DB, Upchurch BR (2013). Multiple pancreatic pseudocysts treated with endoscopic transpapillary drainage. Clin Pract.

[6] Bhasin DK, Rana SS, Udawat HP (2006). Management of multiple and large pancreatic pseudocysts by endoscopic transpapillary nasopancreatic drainage alone. Am J Gastroenterol.

[7] Musana KA, Yale SH, Abdulkarim A, Rall CJ (2004). Successful endoscopic treatment of mediastinal pseudocysts. Clin Med Res.

[8] Sharma SS, Singh B, Jain M (2016). Endoscopic management of pancreatic pseudocysts and walled-off pancreatic necrosis: a two-decade experience. Indian J Gastroenterol.

